# The Effect of Supplementation with Some Essential Oils on the Mobility and the Vitality of Human Sperm

**DOI:** 10.1155/2019/4878912

**Published:** 2019-05-14

**Authors:** Modou M. Mbaye, Bouchra El Khalfi, Boutaina Addoum, Papa D. Mar, Brahim Saadani, Noureddine Louanjli, Abdelaziz Soukri

**Affiliations:** ^1^Physiopathology, Molecular Genetics and Biotechnology Laboratory, Ain Chock Faculty of Science, Biology and Center for Health Research, Hassan II University of Casablanca, Morocco; ^2^Laboratory of Medical Analyses, Reproductive Biology, Labomac, Casablanca, Morocco; ^3^In Vitro Fertilization Center IRIFIV, IRIS Clinic, Casablanca, Morocco

## Abstract

The objective of this work is to study the improvement effect of some essential oils of sage (*Salvia officinalis*), oregano (*Origanum vulgare*), and eucalyptus (*eucalyptus globulus*) on the physiological parameters characterizing the quality of human sperm (mobility and vitality). We find natural biomolecules to improve sperm quality to increase the chances of success of very low in vitro fertilization (IVF) that stagnate around 20%. Sperm samples were mixed with different concentrations of essential oils. The effect of these essential oils on the motility and vitality of spermatozoa has been analyzed. The mobility was determined using a Computer Assisted Sperm Analysis (CASA). In the other side, the evaluation of sperm vitality was performed by staining eosin 2% and the microscopic examination is carried out via optical microscope. A drop of sperm will be mixed with a drop of eosin solution 2%, spread between the slip and coverslip, then allowed to air dry, and examined under a microscope. A significant improvement in the mobility and vitality of human spermatozoa has been noted with oregano. Eucalyptus after 10 min of exposure also significantly improves the mobility and vitality of the spermatozoa. Sage does not improve mobility for these incubation times but significantly improves vitality.

## 1. Introduction

Infertility is one of the new plagues of the 21st century and more and more couples are affected each year. The difficulties in obtaining a pregnancy were most often attributed to women. But today, male infertility alone or not is present in more than 50% of couples' infertility [1].

Male infertility is reflected in 61% of cases by quantitative (sperm count) and/or qualitative (decreased mobility, vitality and sperm morphology) abnormalities [2].

In our study, we were interested in qualitative anomalies including mobility and vitality.

Because the importance of these two parameters on the chances of conceiving in vitro fertilization (IVF) or naturally is no longer to be demonstrated [3]. They would play an important role in the penetration of the spermatozoon into the cumulus oophorus [4] but also in the processes involved in fertilization [5].

The major causes of decreased mobility and consequently vitality can be due either to the presence of flagellar alteration [6] or secondary necrozoospermia that may be due to genital infection [7], oxidative stress [8], the presence of antispermatic antibodies [9], altered ATP production [10], toxic exposure [11], or abnormalities in epididymal transport of spermatozoa [12].

In order to improve the low success rate of IVF results stagnating at around 20%, the development of new sperm quality improvement drugs (mobility and vitality) through plants is an attractive proposition. It is in this context that we have tested the in vitro effect of the essential oils of oregano, sage, and eucalyptus on the mobility and vitality of spermatozoa.

## 2. Materials and Methods

### 2.1. Chemicals Products

All the chemical products used in this study were purchased from Sigma-Aldrich (St Louis, MO, USA).

### 2.2. Plant Material

Three Essential oils (Eos) were studied; they were obtained directly from leaves of different vegetal species, as shown in Table 1. Plant material was harvested randomly, then washed, and dried in a well-ventilated place at room temperature for ten days before their use according to the method of Sabir et al. [13]. The samples were then isolated from each other's specimens and conserved for extraction.

### 2.3. Essential Oil Extraction

Essential oils were obtained by 3.5 h hydrodistillation using the standard Clevenger apparatus according to the method of Sabir et al. [13].The oils were extracted from the distillate with hexane and dehydrated by passing through anhydrous sodium sulfate. After filtration, the solvent was removed by distillation under reduced pressure in a rotary evaporator at 35°C, and the pure oils stored in an amber vial kept under refrigeration (4°C), until their use [13]. The nonlethal concentrations of the essential oils were determined by the dilution technique in series according to the method of Mar et al. [14].

### 2.4. Semen Sample Collection

This study was carried out at laboratory of medical analysis, and Biology of Reproduction, “Labomac”, Casablanca, Morocco. We established two study subgroups: 22 samples which were collected from men diagnosed as normozoospermic (concentration ≥ 20 x 106 / ml, progressive motility is ≥ 32%) and 22 samples which were collected from men diagnosed as asthenozoospermia (≥ 20 × concentration x 106 / ml, progressive motility is <32%). An informed consent was obtained from all the patients included before using their sperm in this study. Then the samples were collected in sterile and labeled containers through the method of masturbation after 3 to 4 days of abstinence period.

For liquefaction step the samples were stored at 37°C under 5% of CO_2_ until their examination. We checked at an interval time of 10 min until the liquefaction was made. The microscopic analysis was performed according to standards and guidelines of the World Health Organization (WHO) [15].

### 2.5. Semen Treatment

**Figure 1:**
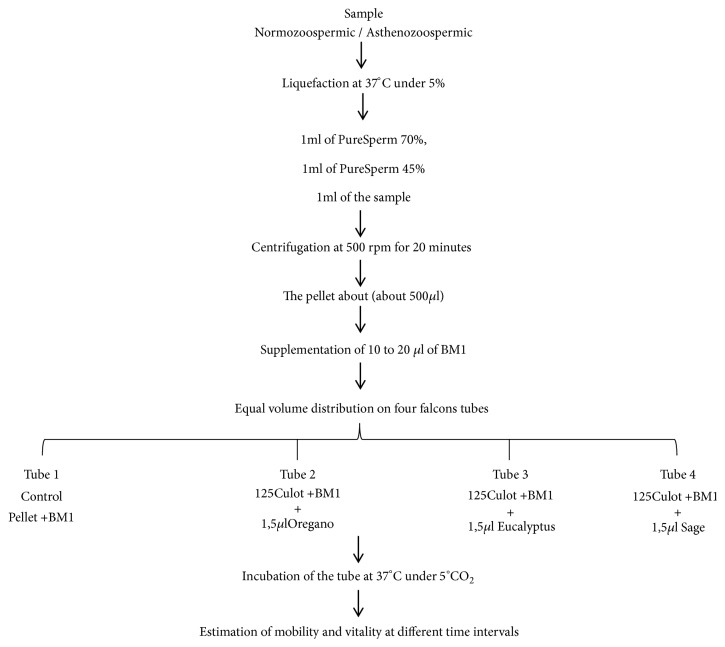
Sample processing treatment methodology.

After one hour of semen production, a routine sperm analysis was carried out in order to determine the sperm count, the motility, and the vitality using Makler counting chamber of 20 *μ*m. The pretreatment of the sperm were performed by the optimization technique of density gradient. Thus 1ml of pure sperm 70%, 1ml of pure sperm 45%, and 1ml of sperm sample were treated, respectively; thereafter they were added in a falcon tube of 10 ml, and centrifuged at 500 rpm for 20 minutes. The remaining sperm fraction at the bottom of the tube for either the normozoospermic or asthenozoospermic sample was completed with 0.5 to 1 ml of BM1 and then the set was divided into four equal aliquots in 10ml falcon's tubes. The first tube containing that the sperm fraction added to BM1 was incubated at 37°C under 5% CO_2_ as a control, the other three remaining tubes were incubated at 37°C under 5% CO_2_, respectively, with 1.5 *μ*l of each essential oil namely oregano, eucalyptus, and sage (Figure 1).

### 2.6. Analysis of the Sperm Motility

The effect of sage, oregano, and eucalyptus oils on the mobility of human spermatozoa was monitored at different times of incubation: 0, 5, 10, 15, 20, 25, and 30.35 min at 37°C under 5% of CO_2_. The protocol consists to put down an aliquot of 10 *μ*l of the mixture (sperm / oils, control) on a Makler counting chamber of 20 *μ*m. Then we observed with CASA: Computer Assisted Semen Analysis Hamilton-Thorne version 10 HTM IVOS Analyzer (Hamilton-Thorne Biosciences, Beverly, MA, USA), immediately after we lay down the mixture [16].

### 2.7. Analysis of the Sperm Vitality

The evaluation of sperm viability was performed with 2% eosin staining [17]. From there, we proceeded to the following protocol: A drop of sperm and a drop of 2% eosin solution were both mixed and spread between the microscopic slip and the coverslip, after the lay down of the mixture we wait for the air drying of the preparation; then we observed under an optical microscope [18]. Test is carried out immediately after the contact between the spermatozoa and different concentrations of EOs (t = 0 min), at a range of exposure times 5, 10, 15, 20, 25, 30, and 35 min and we incubated the preparation at 37°C under 5% of CO_2_ [19].

### 2.8. Statistical Analysis

The data obtained during our experiment are the subject of a statistical study. The results of supplementation with EOs on the physiological parameters (motility and the vitality) of human sperm were performed by the Student's t-test (Test t). All the graphs and the histograms represented in this paper were carried out using the software: GraphPadPrism7.

## 3. Results

In order to investigate the in vitro effect of EOs on physiological parameters (mobility and vitality) of human sperm and to choose the ideal exposure time of each HE with sperm, a test was performed on four semen samples classified as normal according to the guidelines of the World Health Organization at different exposure times (0, 5, 10, 15, 20, 25, 30, and 35 min) between sperm and each EO. The average of the spermatic parameters (mobility and vitality) of the four samples showed that essential oils improve mobility and vitality of human sperm. Based on our finding the most interesting values were observed with oregano at 5min of incubation with 73 ± 0.07 for mobility and 74 ± 0.06 for vitality, eucalyptus, and sage at 10 min provide their greatest values, respectively: 72 ± 0.05 and 58 ± 0.08 for mobility and 72 ± 0.13 and 66 ± 0.03 for vitality. Therefore, the sampling was extended to 44 samples, divided into two subgroups: the first group included 22 of men with normozoospermic profiles (concentration ≥20 × 106 / ml, progressive motility ≥ 32%) and the second group is formed by 22 of men with asthenozoospermic profiles (concentration ≥20 × 106 / ml, progressive motility <32%).

### 3.1. Effects of Essential Oils on the Motility of Spermatozoa

**Figure 2:**
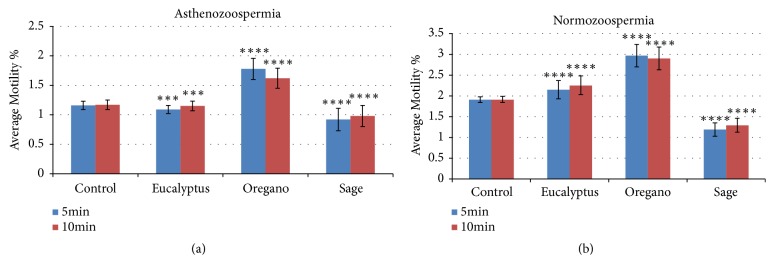
The in in vitro effect of EOs on the proportion of spermatozoa with fresh mobility ≥ 32%. The dilution of EOs was prepared in a solution of sterile agar at 0.2% (w / v) for a range of concentrations: 10^−1^, 10^−2^, 10^−3^, and 10^−9^. The motility percentage of each aliquot was measured immediately after the addition of the HE (t = 5 min) and 10 minutes later (t = 10 min). The data are expressed as mean ± standard deviation (n = 5). (*∗*) indicates “p” values: *∗* significant (p <0.05); *∗∗* highly significant (p <0.01); *∗∗∗* highly significant (p <0.001).

The effect of the EOs of sage, eucalyptus, and oregano on the mobility of human sperm is reported in Figure 1; it is based on the determination of the motile proportion of spermatozoa. As shown in Figure 1, a significant ameliorative effect of mobility after the supplementation with essential oils was observed; it exhibited a dose-dependent effect. Nevertheless, another potent effect was observed after an exposure time of 10min with eucalyptus and normal sperm (Figure 2). The acute effect of the EOs was observed with oregano after 5 and 10 min of incubation as well on the normal sperm as on the asthenozoospermia.

The most significant improvement on the mobility was observed after an exposure time of 5 min between sperm and oregano (Figure 2). However, with sage no improvement effect was observed as well from 5min to 10min.

### 3.2. The Effects of Essential Oils on the Viability of Spermatozoa

**Figure 3:**
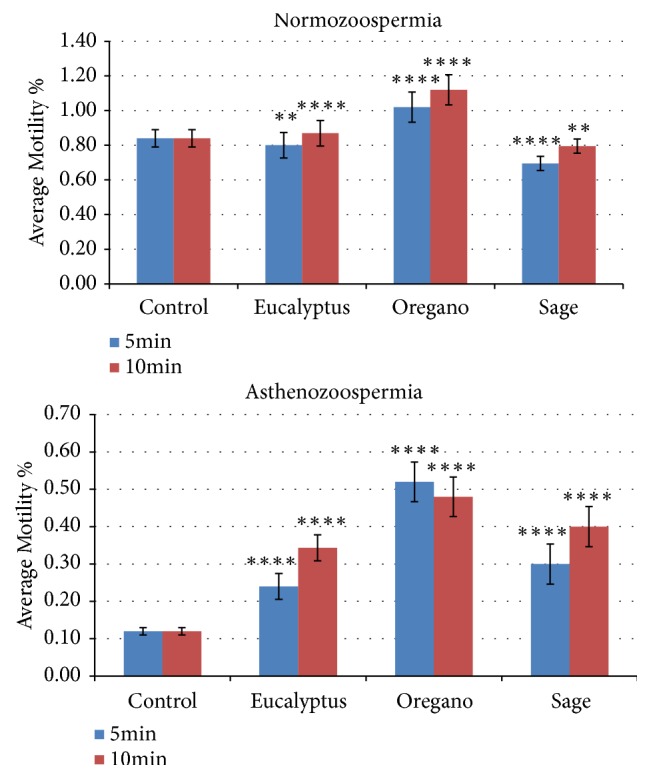
The in vitro effect of EOs on the proportion of spermatozoa with a vitality ≥ 58%. We prepared the dilution of EOs in a sterile solution of agar at 0.2% (w / v) for a range concentration of 10^−1^, 10^−2^, 10^−3^, and 10^−9^. The motility percentage of each aliquot was measured immediately after the supplementation with the HE (t = 5 min) and 10 minutes later (t = 10 min). The data are expressed as mean ± standard deviation (n = 5). (*∗*) indicates “p” values: *∗* significant (p <0.05); *∗∗* highly significant (p <0.01); *∗∗∗* highly significant (p <0.001).

The effect of sage, eucalyptus, and oregano oils on the vitality of human sperm was determined by using the 2% eosin staining method, and the results of this experiment are illustrated in Figure 2. To assess the semen vitality we used the same preparation of the mobility test. According to the results reported in Figure 2 as a function of the same exposure time; it clearly showed a significant decrease in the number of viable spermatozoa. In this experiment we used as a control the asthenozoospermia samples after a time exposure of 5 and 10 min. Based on our data (Figure 3) we can deduce that the vitality of the spermatozoa after the supplementation with the different EOs did not tumble (Figure 3). In the other side the vitality was more significant with the oregano. Even if the sage does not improve the mobility after 5 min or after 10min, it is possible to make a decrease in order to improve the vitality of the spermatozoa.

## 4. Discussion

The results of the study reveal that incubation at 37°C under 5% CO_2_ of human sperm with essential oils of oregano, sage, and eucalyptus improves the mobility and vitality of spermatozoa.

Oregano at 5 min incubation gave the most interesting values of mobility and vitality respectively of 73±0.07% and 74±0.07%. However, the latter drop slightly after 10 min incubation at 37°C under 5% CO_2_.

Eucalyptus gives values at 5 and 10min incubation at 37°C under 5% CO_2_ in percentages of the order of 72±0.05% and 72±0.13%. These values remain lower than those of oregano.

On the other hand, sage at 5 and 10 minutes incubation at 37°C under 5% CO_2_ records the lowest values compared to the controls and to the other two essential oils.

The exact mechanism, by which essential oils improve the mobility and vitality of human sperm, remains unclear and of great interest [20]. De Iuliis, G. N., et al. argue that mobility and vitality can be damaged by several factors free radicals such as reactive oxygen species (ROS). Reducing ROS can help to treat the mobility and vitality of human sperm cells [21].

Studies have shown that the essential oils of oregano, eucalyptus, and sage contain important free radical sensors such as saponins[22], alkaloids[23], flavonoids[24], and phenolic compounds [24] that may have a direct effect on ROS activity by trapping them or an indirect effect by increasing the production of intracellular antioxidant enzymes.

It is worth remembering following the comparison of the effects of the three essential oils of oregano, eucalyptus, and sage show that oregano gives the best values of mobility and vitality followed by eucalyptus. On the other hand, sage gives interesting values but remains lower than the values of the controls.

## 5. Conclusion

The present study highlights the valuation of medicinal plants through the demonstration of their improving properties of spermatic quality by evaluating in vitro their impact on the mobility and vitality of human sperm. In this evaluation of the effect of these EOs on human sperm, the results allowed us to observe an increase in sperm quality (mobility and vitality) by EO. Oregano after 5min has significantly improved the motility and vitality of human spermatozoa. Eucalyptus after 10 min of exposure also significantly improves the mobility and vitality of the spermatozoa. Sage, on the other hand, does not improve mobility at these incubation times but significantly improves vitality. Based on the results, it can be concluded that these EOs can be a safe therapeutic alternative for the management of motility and vitality dysfunction.

## Figures and Tables

**Table 1:** Plant species used in the experiment for EOs extraction.

Plant family	Scientific name	Local name	Collection site	Plant Part
Myrtaceae	*Eucalyptus Globulus*	Fliyou	Fes	Leaves
Lamiaceae	*Oreganum Vulgare*	Fliyou	Fes	Leaves
Lamiaceae	*Salvia Officinalis*	Fliyou	Fes	Leaves

## Data Availability

The data used to support the findings of this study are available from the corresponding author upon request.

## References

[1] Comhaire F. H., Rowe P. J., Farley T. M. M. (1986). The effect of doxycycline in infertile couples with male accessory gland infection: a double blind prospective study. *International Journal of Andrology*.

[2] Bonde J. P., Giwercman A., Ernst E. (1996). Identifying environmental risk to male reproductive function by occupational sperm studies: Logistics and design options. *Occupational and Environmental Medicine*.

[3] Lewis S. E. (2007). Is sperm evaluation useful in predicting human fertility?. *Reproduction*.

[4] Palomo M. J., Mogas T., Izquierdo D., Paramio M. T. (2010). The influence of sperm concentration, length of the gamete co-culture and the evolution of different sperm parameters on the in vitro fertilization of prepubertal goat oocytes. *Zygote*.

[5] Auger J., Eustache F., Ducot B. (2000). Intra- and inter-individual variability in human sperm concentration, motility and vitality assessment during a workshop involving ten laboratories. *Human Reproduction*.

[6] Haidl G., Badura B., Hinsch K.-D., Ghyczy M., Gareiß J., Schill W.-B. (1993). Disturbances of sperm flagella due to failure of epididymal maturation and their possible relationship to phospholipids. *Human Reproduction*.

[7] Souho T., Benlemlih M., Bennani B. (2015). Human papillomavirus infection and fertility alteration: A systematic review. *PLoS ONE*.

[8] Gomez E., Buckingham D. W., Brindle J., Lanzafame F., Irvine D. S., Aitken R. J. (1996). Development of an image analysis system to monitor the retention of residual cytoplasm by human spermatozoa: correlation with biochemical markers of the cytoplasmic space, oxidative stress, and sperm function. *Journal of Andrology*.

[9] Lee R., Goldstein M., Ullery B. W. (2009). Value of serum antisperm antibodies in diagnosing obstructive azoospermia. *The Journal of Urology*.

[10] Folgerø T., Bertheussen K., Lindal S., Torbergsen T., øian P. (1993). Andrology: Mitochondrial disease and reduced sperm motility. *Human Reproduction*.

[11] Wilton L. J., Temple-Smith P. D., Baker H. W. G., De Kretser D. M. (1988). Human male infertility caused by degeneration and death of sperm in the epididymis. *Fertility and Sterility*.

[12] Axnér E., Linde-Forsberg C., Einarsson S. (1999). Morphology and motility of spermatozoa from different regions of the epididymal duct in the domestic cat. *Theriogenology*.

[13] Sabir A., El-Khalfi B., Errachidi F. (2017). Evaluation of the potential of some essential oils in biological control against phytopathogenic agent pseudomonas syringae pv. tomato dc3000 responsible for the tomatoes speck. *Journal of Plant Pathology & Microbiology*.

[14] Cooper T. G., Noonan E., von Eckardstein S. (2009). World Health Organization reference values for human semen characteristics. *Human Reproduction Update*.

[15] Mar P. D., El Khalfi B., Soukri A. (2018). Protective effect of oregano and sage essentials oils against the effect of extracellular H2O2 and SNP in Tetrahymena thermophila and Tetrahymena pyriformis. *Journal of King Saud University - Science*.

[16] Hirano Y., Shibahara H., Obara H. (2001). Relationships between sperm motility characteristics assessed by the computer-aided sperm analysis (CASA) and fertilization rates in vitro. *Journal of Assisted Reproduction and Genetics*.

[17] Björndahl L., Söderlund I., Kvist U. (2003). Evaluation of the one-step eosin-nigrosin staining technique for human sperm vitality assessment. *Human Reproduction*.

[18] Amelar R. D., Dubin L., Schoenfeld C. (1973). Semen analysis. An office technique. *Urology*.

[19] Yeung C.-H., Cooper T. G. (2010). Sperm quality and function tests. *Andrology*.

[20] De Iuliis G. N., Newey R. J., King B. V., Aitken R. J. (2009). Mobile phone radiation induces reactive oxygen species production and DNA damage in human spermatozoa *In vitro*. *PLoS ONE*.

[21] Rezvanfar M. A., Sadrkhanlou R. A., Ahmadi A. (2008). Protection of cyclophosphamide-induced toxicity in reproductive tract histology, sperm characteristics, and DNA damage by an herbal source; evidence for role of free-radical toxic stress. *Human & Experimental Toxicology*.

[22] Singh S., Lata S., Tiwari K. N. (2015). Antioxidant potential of phyllanthus fraternus webster on cyclophosphamide induced changes in sperm characteristics and testicular oxidative damage in mice. *Indian Journal of Experimental Biology (IJEB)*.

[23] Khaki A., Ghanbari Z., Ghanbari M. (2011). Anti-oxidative effects of citro flavonoids on spermatogenesis in rat. *African Journal of Pharmacy and Pharmacology*.

[24] de Lamirande E., Gagnon C. (1992). Reactive oxygen species and human spermatozoa: II. Depletion of adenosine triphosphate plays an important role in the inhibition of sperm motility. *Journal of Andrology*.

